# Dr. G. H. Moody

**Published:** 1911-09

**Authors:** 


### Dr. G. H. Moody.

The Neurological Section of the A. M. A. honored the Southwest by selecting as Vice Chairman Dr. G. H. Moody, of San Antonio, Texas. Dr. Moody was educated at the Southwestern University, Georgetown, Texas, and, in 1896, received his M. D. from Tulane. He took post-graduate work in New York, London and Vienna. For six years he was active staff member of the hospitals for Insane at San Antonio and Austin, Texas. In 1903 Dr. Moody founded the Moody Sanitarium for Mental and Nervous Diseases. This institution has prospered and grown with Dr. Moody till both are essentials of the profession of the great State of Texas.

Dr. Moody served the Texas State Association twice as Chairman of the Neurological Section. He is ex-President of his home county society, for the Fifth District Society, and in 1909 was elected President of the Medical Society of the Southwest. We congratulate Dr. Moody in his official step up into the National Association, for his work warrants this recognition. Quiet, unobtrusive, modest, discovered by his merits only, Dr. Moody has risen to an enviable position among the leading neurologists of America.—S. G. B., in *The Medical Herald.*

